# Endothelial glycocalyx as an important factor in composition of blood‐brain barrier

**DOI:** 10.1111/cns.13560

**Published:** 2020-12-30

**Authors:** Fangfang Zhao, Liyuan Zhong, Yumin Luo

**Affiliations:** ^1^ Institute of Cerebrovascular Disease Research and Department of Neurology Xuanwu Hospital of Capital Medical University Beijing China; ^2^ Beijing Key Laboratory of Translational Medicine for Cerebrovascular Diseases Beijing Geriatric Medical Research Center Beijing China; ^3^ Beijing Institute for Brain Disorders Capital Medical University Beijing China

**Keywords:** blood‐brain barrier, endothelial glycocalyx, heparan sulfate, permeability

## Abstract

The blood‐brain barrier is a dynamic and complex neurovascular unit that protects neurons from somatic circulatory factors as well as regulates the internal environmental stability of the central nervous system. Endothelial glycocalyx is a critical component of an extended neurovascular unit that influences the structure of the blood‐brain barrier and plays various physiological functions, including an important role in maintaining normal neuronal homeostasis. Specifically, glycocalyx acts in physical and charge barriers, mechanical transduction, regulation of vascular permeability, modulation of inflammatory response, and anticoagulation. Since intact glycocalyx is necessary to maintain the stability and integrity of the internal environment of the blood‐brain barrier, damage to glycocalyx can lead to the dysfunction of the blood‐brain barrier. This review discusses the role of glycocalyx in the context of the substantial literature regarding the blood‐brain barrier research, in order to provide a theoretical basis for the diagnosis and treatment of neurological diseases as well as point to new breakthroughs and innovations in glycocalyx‐dependent blood‐brain barrier function.

## Graphical abstract

Endothelial glycocalyx as an important factor in composition of blood‐brain barrier and significance.

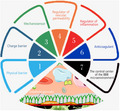

## 1 INTRODUCTION

The blood‐brain barrier (BBB) is a dynamic and complex neurovascular unit, which protects the brain parenchyma from the influence of circulatory factors and regulates and maintains the internal environment stability of central nervous system. A stable internal environment is necessary for normal nerve function.^1^, ^2^ Damage to the blood‐brain barrier is a major part of the pathophysiological process of many nervous system diseases, such as multiple sclerosis, stroke, Alzheimer's disease, vascular dementia, cerebral small vessel disease, traumatic brain injury, and epilepsy.^3^, ^4^, ^5^, ^6^, ^7^, ^8^, ^9^, ^10^, ^11^, ^12^

**FIGURE 1:**
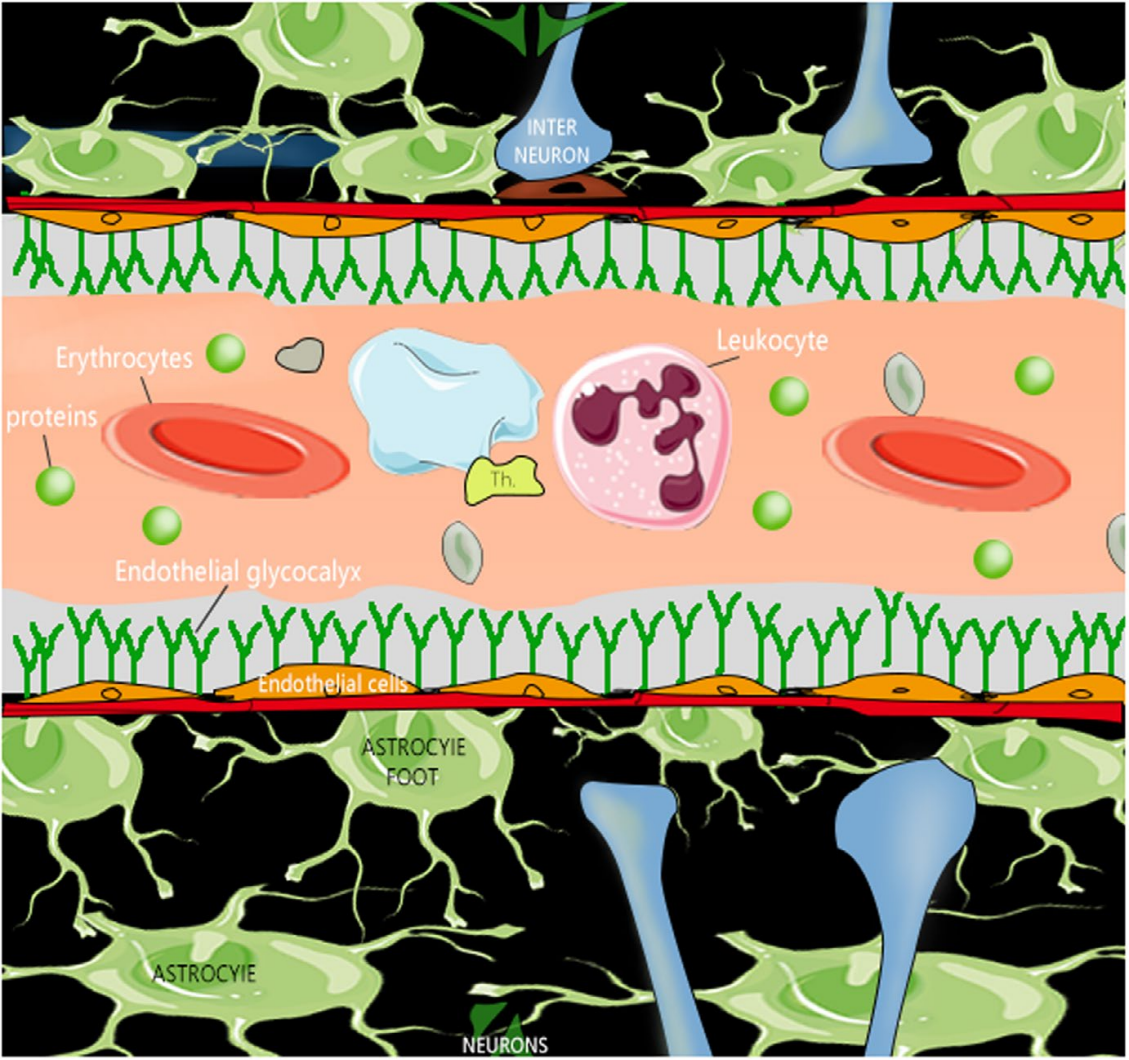
Diagram of the relationship between glycocalyx and the blood‐brain barrier

Recently, endothelial glycocalyx (EG) has been defined as a component of the expanded neurovascular unit, an important physiological structure that maintains proper neuronal homeostasis.^13^ In its simplest sense, EG is related to maintaining the integrity of the blood vessel wall. However, the more nuanced functions of EG have been greatly ignored for a long time. In 1966, Luft first observed that EG appeared as an irregular fluffy layer under an electron microscope, extending only 20 nanometers into the vascular cavity.^14^ Thirty years later using intravital microscopy, Vink and Duling observed that EG appears as a thicker structure,^15^, ^16^ consistent with our current knowledge that EG forms a protective "jelly‐like" layer covering the endothelial cells lining the surface of the vascular lumen. The volume of human glycocalyx is estimated to be about 1700 ml.^17^ The relationship between glycocalyx and BBB is shown in Figure 1.

As a surface coating, EG forms a barrier between blood and blood vessels. The damage of EG seems to be the first step in BBB dysfunction,^18^ which is an important aspect of many diseases.^19^, ^20^ Although EG does appear to be an important regulator of BBB functions, there are relatively few studies on the relationship between EG and BBB at present. Therefore, by reviewing the potential significance of EG in the BBB, this review aims to find new breakthroughs and innovations in the research of BBB for hundreds of years and to provide a theoretical basis for the diagnosis and treatment of neurological diseases.

## 2 THE STRUCTURE OF EG

**FIGURE 2:**
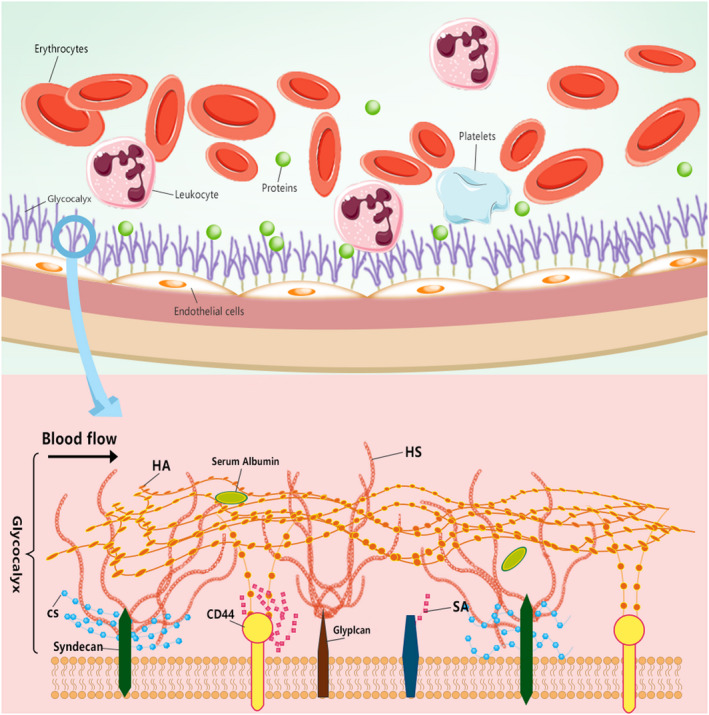
Molecular structure pattern of glycocalyx. HS: heparan sulfate, CS: chondroitin sulfate, HA: hyaluronic acid, SA: sialic acid

Endothelial glycocalyx, also referred to as the polysaccharide coating or matrix, is located on the apical surface of vascular endothelial cells and consists of a layer of light blue substance with a thickness of about 20 nm. At present, it is generally believed that EG is a highly negatively charged surface layer on endothelial cells, mainly composed of proteoglycan (PG) and its linked glycosaminoglycan (GAG) chains.^21^ PG is considered as the most important structural molecule in EG, consisting of a core protein connected to a mucopolysaccharide chain.^22^ The core protein is an important part of supporting the connection between GAG chains and endothelial cells, and mainly includes multiligand glycans and phosphoinositol glycans, which are closely connected with endothelial cells through transmembrane domains and glycosylated phosphoinositide anchors, respectively.^23^ As the most abundant component in EG, GAG mainly includes heparan sulfate (HS), chondroitin sulfate (CS), keratan sulfate (KS), and hyaluronic acid (HA) (Figure 2). HS is the main part of the inner membrane surface, accounting for 50%‐90% of GAG. HA is a network structure with long molecules woven on the surface of the endothelial lumen. CD44 is the main receptor of HA. In contrast, HS is a shorter molecule, positioned perpendicular to the cell surface. On the surface of the endothelial lumen, HA and HS partially overlap. HS plays a major role in mechanical sensing, and HA plays a major role in molecular sieve.^24^ Many glycoproteins end with sialic acid (SA). The heparan sulfate proteoglycans (HSPG) on the lumen side of vascular endothelial cells mainly include the Syndecan family and the Glypican family. The whole EG is regarded as a highly dynamic structure that is variable in form and structure and constantly renews itself.^25^

## 3 THE SIGNIFICANCE OF EG ON THE BLOOD‐BRAIN BARRIER

### 3.1 Physical barrier

Of all of the many functions of EG, the most important role is its contribution to the physical barrier between plasma and endothelial cells. The formation of EG at the endothelial lumen reduces the chance of plasma components entering the endothelial surface,^26^ and at the same time blocks blood cells and other harmful components of plasma.^27^, ^28^, ^29^ Molecules to which EG functions as a barrier to endothelial cells include adhesion molecules (ICAM/VCAM), von Willebrand factor, antithrombin, tissue factor pathway inhibitors, NO synthase (NOS), and extracellular superoxide dismutase. In addition, leukocytes, erythrocytes, and platelets cannot easily penetrate EG; a complete and stable EG inhibits the interaction not only between molecules and endothelial cells, but also between plasma cells and endothelial cells. The adhesion between white blood cells and endothelial cells is a receptor‐mediated process, and the adhesion receptors on the surface of endothelial cells are located in EG.^30^ In the disease state, EG reduction can promote leukocyte adhesion to endothelial cells by exposing adhesion receptors.^31^, ^32^ Intact EG can also reject red blood cells from the endothelium. In the microcirculation, a red blood cell repelling zone has been observed next to the endothelium; this repelling zone decreases in correlation with the decomposition of EG induced by a light dye.^33^ Similarly, under control conditions, platelets seldom interact with endothelium, but the platelet‐vessel wall interaction increases after removing part of EG by injecting oxidized low‐density lipoprotein (ox‐LDL). Degradation of EG in inflammatory or ischemic disease states leads to the interaction between blood cells and endothelial cells,^15^, ^34^, ^35^ eventually resulting in endothelial dysfunction and damage to the blood‐brain barrier. In addition, EG as a physical barrier can prevent BBB dysfunction caused by oxidative stress. By studying the structure and functions of EG, we can lessen BBB dysfunction by shoring up the protective barrier provided by EG.

### 3.2 Charge barrier

The high sulfation of GAG gives EG a net negative charge. As a charge and size barrier, the negatively charged EG promotes (or prevents) protein diffusion to the surface of endothelial cells, a level of control essential for the integrity of the BBB. The negative charge depends on the side chain structure of GAG, and its pattern is affected by time, physiological, and pathophysiological stimulation. A large amount of sulfate residues on sugar chains, especially heparin sulfate, leads to negative charges on EG, leading to the facilitation of positive charges binding and exclusion of negatively charged molecules.^36^ The charged network of EG functions like a giant molecular sieve, which can resist negatively charged molecules. Due to the charge characteristics of EG, it is also semipermeable. The negatively charged EG surface also forms an electrostatic barrier for plasma cells and proteins.^37^

### 3.3 Mechanosensor

The vascular endothelium is affected by shear stress and blood pressure. EG plays an important role in the mechanical transformation under shear stress. Studies have shown that GAG side chains, such as HS, are important shear sensors.^28^ As integral elements of EG, Syndecans and Glypicans convert mechanical stimuli into intracellular signals that control endothelial structure and functions. Syndecan‐1 can regulate the rearrangement of the cytoskeleton molecules such as tubulin, alpha‐actinin, and EG, leading to smooth muscle cell relaxation and vasodilation. In 2003, Weinbaum et al. described an EG arrangement as "shrub‐like" proteoglycan clusters projected from the anchor points of the endothelial cell cytoskeleton. The force generated by the mechanical deformation of the entire bush deforms the cytoskeleton. This increases the expression of nitric oxide synthase protein in endothelial cells, which catalyzes the production of NO and increases hydraulic conductivity. Therefore, EG is considered to be an important sensor that converts fluid shear stress into cell signals (ie, mechanical transduction). EG can be degraded by HS or hyaluronidase (HAase),^28^, ^38^ which blocks the production of shear‐dependent NO, leading to vasomotor dysfunction.

### 3.4 Regulator of vascular permeability

Dysfunction of the BBB results in increased vascular permeability,^39^ which causes fluid and macromolecules to pass through the BBB into the central nervous system.^40^ Traditionally, tight junctions and adherent junctions between endothelial cells have been considered the main determinants of BBB functions.^41^ However, in the past few years, the endothelial EG layer has been recognized as a key regulator of permeability. The mechanisms of EG regulating BBB permeability are as follows. (a) EG plays an important role in maintaining the permeability of BBB due to its matrix structure and negative charge. Yuan et al. found that the permeability of positively charged materials is four times that of negatively charged materials.^42^ (b) EG may mediate the transmembrane transport of endothelial cells. For example, the negative charge of EG plays a key role in the transmembrane transport of specific molecular substances that vary in diameter.^43^ Partial removal of EG in the myocardial capillaries of rats leads to a loss of the barrier function of blood vessels and an increase in permeability, which results in the occurrence of myocardial edema.^44^ The research of Rehm et al.^44^ and Jacob et al.^45^ also emphasizes the importance of endothelial EG in controlling colloidal and liquid extravasation. Kai et al. also demonstrated that the sialic acid residues in endothelial EG are key regulators of the microvascular permeability of water and albumin.^46^

When Starling put forward the traditional concept of capillary liquid exchange in 1896, EG was not factored into the model.^47^ The Starling equation describes the balance between the hydrostatic pressure and osmotic pressure acting on the microvascular lumen. Traditionally, the main factor thought to regulate penetration is the tight junction between endothelial cells. Although the Starling principle is not entirely wrong, it is incomplete. Adamson et al. observed that the increased interstitial osmotic pressure in rat postcapillary venules resulted in only a small increase in fluid filtration.^48^ The colloidal imaging of albumin distribution *in vivo* also showed the lack of EG regions. These observations indicated that the main osmotic pressure gradient opposite to fluid filtration begins with EG, but the effect of interstitial protein concentration was small. Michel and Levick proposed a “modified” Starling principle suggest that Starling force should only be applied to EG, which is now considered as a molecular sieve for plasma proteins.^49^ When EG was removed experimentally, vascular permeability increased significantly.^50^ The above observations indicate that the EG layer, which reduces the permeability of the BBB, can protect blood vessels, thus reducing the dysfunction of the blood‐brain barrier. In addition, the function of EG molecules can also be adjusted to increase the BBB permeability, so as to achieve the therapeutic effect.

### 3.5 Regulator of inflammation

Studies have shown that HS in EG can act as a ligand for L‐selectin and participates in the turnover and shift of leukocytes in blood vessels. HS can adjust the concentration gradient of chemokines, such that endothelial cells and leukocytes adhere closely. Wang et al. found that knocking out the HS gene in rats reduces the accumulation and adhesion of vascular endothelial chemokines.^51^ In addition, recent studies have found that EG shedding caused by inflammation further promotes monocyte adhesion and the infiltration of lipid‐retained macrophages.^52^ Similar to antioxidants in plasma, proinflammatory cytokines and immune cells can be buffered by cellular EG.^53^ If EG is eventually destroyed by proinflammatory cytokines, endothelial cells become a prime location for immune cells to adhere and infiltrate, which itself promotes further inflammation. EG also regulates inflammation by binding to cytokines and weakening the binding of cytokines to cell surface receptors.

### 3.6 Anticoagulant

As an important part of BBB, vascular EG can prevent the direct contact between endothelium and blood cells under physiological conditions, thus avoiding thrombosis. The main mechanisms include the following: (a) Antithrombin III combines with HS on EG to enhance its anticoagulant; (b) thrombomodulin can bind to CS and convert thrombin into an activator of protein C pathway, thus forming anticoagulant pathway; (c) tissue factor pathway inhibitor (TFPI) is an effective inhibitor of FVIIa and FXa in the coagulation pathway, which mainly achieves anticoagulation through the interaction between HS and EG.^54^ In addition, many anticoagulant molecules exist in EG itself, providing an endothelial cell‐based anticoagulant defense.^55^ EG also can interact with antithrombin III, heparin cofactor II, thrombomodulin, and TFPI to achieve anticoagulation. Antithrombin III is a powerful thrombin inhibitor, which binds to specific parts of HS to enhance its anticoagulant activity. Heparin Cofactor II is a thrombin specific protease inhibitor, which is activated by KS in EG.^56^ TFPI is an effective inhibitor of FVIIa and FXa, which may bind to EG through HS sulfate.^57^ In addition, the uptake and degradation of the TFPI‐FXa complex depends on the HS in EG.^56^ Together, these molecules in EG contribute to the inhibition of coagulation by endothelial cells.

### 3.7 EG as the control center of the BBB microenvironment

Glycosaminoglycan plays an important role in the control of BBB microenvironment. The diversity of GAG sugar chains comes from its extension, elongation, and sulfation, all of which create a heterogeneous surface on which many molecules from the plasma may dock. Plasma‐derived molecular docking can affect the local environment in several ways: (a) The binding of receptors, enzymes, and their ligands to EG causes the local concentration of these substances to rise, which allows corresponding signal transduction or enzyme modification to be possible. Fibroblast growth factor signal is mediated in this way and is completely dependent on the interaction of ligands and receptors with EG.^58^, ^59^, ^60^ Similarly, EG participates in the lipolysis system by combining lipoprotein lipase and its ligand, low‐density lipoprotein.^61^ (b) The binding of plasma‐derived molecules to EG can cause local concentration gradients, a common differential impacting the process of gene transcription and development regulated by growth factors.^62^ (c) Linking enzymes and their agonists or inhibitors with EG molecule may enable EG have vascular protective functions.

## 4 THE IMPACT OF EG DAMAGE ON BBB

Blood‐brain barrier is a specialized tissue in the brain that strictly controls the transport of metabolites between blood and brain. Full function of the BBB function is essential for brain homeostasis and normal neuronal function. Many neurological diseases are related to the disruption of the BBB. Previously, the function of BBB was thought to depend primarily on vascular endothelial cells and paracellular auxiliary structures,^63^ but more recently, EG is regarded as an important determinant of BBB function.^64^ EG is a fragile layer, and as a part of the BBB, its destruction can cause BBB dysfunction.^65^ BBB dysfunction caused by EG damage can be divided into the following aspects: (a) EG degradation destroys the physical barrier, leading to aggravated inflammation and causing physiological disorders of microcirculation. EG can block blood cells and plasma substances from contacting with endothelial cells. The destruction of the physical barrier afforded by EG can cause blood cells and other harmful components to contact endothelial cells, leading to the increase of local inflammation, edema, platelet aggregation, oxidative stress, and loss of vascular reactivity, ^61^, ^62^ ultimately causing BBB dysfunction. In the hamster cremaster muscle, Vink et al. used oxidized lipoproteins to degrade EG, noting a subsequent increase in the number of platelets that interact with endothelial cells.^56^ Using a similar method, Henry and Duling demonstrated that degrading EG can promote leukocyte‐endothelial cell adhesion.^57^ (b) Endothelial dysfunction increases BBB permeability. When EG exists, endothelial cells proliferate and arrange in the direction of laminar flow, but endothelial cells do not proliferate and align after EG degradation induced by heparinase,^59^ causing endothelium disfunction. The main consequences of EG loss increase BBB permeability, aggravation of edema, and vasodilation dysfunction. Using various enzyme‐mediated degradation technologies to destroy the integrity of EG leads to an increase in the conductivity of water and protein.^38^ Zhu et al. observed the changes of EG and BBB function in a model of cardiopulmonary resuscitation in rats after cardiac arrest, observing that EG degradation aggravates brain damage, increases BBB permeability, and leads to vasogenic brain edema. Additionally, the thickness of EG is negatively correlated with the degree of BBB dysfunction.^20^ Thus, EG protection could reduce brain damage. (c) EG disruption may lead to the release of adhesion molecules. The EG contains many molecules related to BBB integrity, such as vascular cell adhesion molecules (VCAM) and intercellular adhesion molecules (ICAM). When the EG is compromised, adhesion molecules are released and allow for increased adhesion of blood cells such as leukocytes to the cell adhesion molecules.^66^ (d) The shedding of EG leads to the endothelial dysfunction of BBB by disruption of biomechanical responses. Biomechanical disorders elicited by EG shedding enhance edema formation, destroy the interaction between cells and blood vessel walls, and lead to loss of fluid shear stress sensitivity. In the case of continuous shear stress, vascular inflammation occurs, subsequently leading to atherosclerosis.^54^ Based on these observations and functional extrapolation, the impaired EG may have an important impact on the pathophysiological mechanisms of BBB function, and therefore, EG preservation may be a new brain protection strategy.

## 5 MECHANISMS OF EG DAMAGE

**FIGURE 3:**
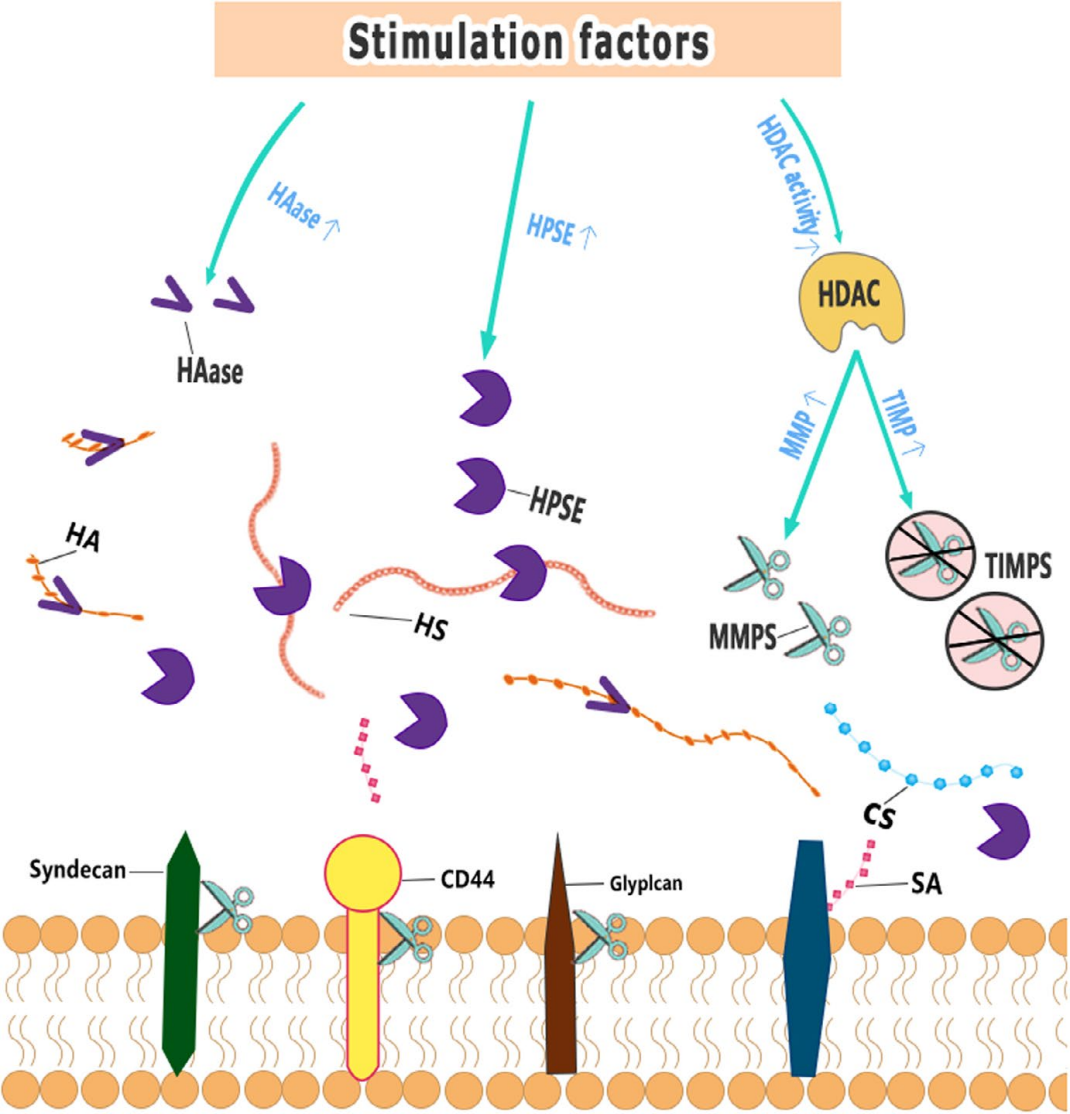
Mechanisms of EG damage. HPSE: Heparanase, HAase: hyaluronidase, MMPS: matrix metalloproteinases, TIMPS: tissue inhibitor of matrix metalloproteinases, HDAC: histone deacetylase, HA: hyaluronic acid, HS: heparan sulfate, CS: chondroitin sulfate, SA: sialic acid

Investigation into the mechanisms of EG damage has been the target of numerous studies, with several studies identifying matrix metalloproteinases (MMPs) as major molecules responsible for EG shedding.^67^ MMP‐2, MMP‐7, and MMP‐9 directly cut CS, MMP‐1 cuts syndecan‐1,^68^ and MMP‐9 is the major shedding enzyme of syndecan‐4.^69^ ADAM17 is also involved in EG degradation by stripping the extracellular domain of syndecan‐4.^70^ Yang et al. showed that ADAM15 causes vascular BBB dysfunction by causing EG degradation. The underlying mechanism involves ADAM15‐mediated CD44 cleavage and release of the extracellular domain of CD44 into the circulation, thereby promoting vascular hyperpermeability^71^ and BBB disruption. Therefore, blocking ADAM15 could be a potential option for maintaining EG integrity. MMPs are regulated by histone deacetylase (HDAC) inhibitor activity. When HDAC is upregulated upon stimulation, MMP expression increases, and tissue inhibitor of matrix metalloproteinases (TIMPs) expression decreases, resulting in accelerated EG degradation in endothelial cells.^72^ Heparanase (HPSE) is the only enzyme known to cleave HS and is another important factor in promoting EG shedding. Studies of HPSE have helped us to understand the catabolic processes involved in the breakdown of HS. Methylation of the HPSE promoter is a potential regulator of HPSE expression.^73^ Recently, the transcription factor SMAD4, a key protein in the TGF‐β signaling pathway, was found to inhibit HPSE expression by binding to the HPSE promoter region.^74^, ^75^ The inhibitory effect of p53 binding to the promoter on HPSE expression also leads to a decrease in HPSE activity, suggesting that p53 is a potent regulator of HPSE expression and that the loss of this protein alone is sufficient to increase HPSE transcriptional levels.^76^ Another enzyme that promotes EG shedding is HAase. HAase cleaves HA. Among them, atherosclerosis is associated with increased activity of HAase.^77^ Mechanisms of EG damage are shown in Figure 3.

Syndecan‐4 is an NF‐κB target gene.^78^ The NF‐κB p65 subunit is deacetylated by SIRT‐1, which prevents nuclear translocation of NF‐κB.^79^ Thus, increased SIRT‐1 activity inhibits NF‐κB‐dependent inflammatory responses, while decreased SIRT‐1 activity enhances NF‐κB signaling. In SIRT1endo‐/‐ mice, NF‐kB nuclear translocation is increased and syndecan‐4 transcription is elevated,^80^ but the extracellular domain of syndecan‐4 is shed from the endothelial surface of SIRT1endo‐/‐ mice, thus depleting the major scaffold component of EG.^81^ The relationship between SIRT‐1 and NF‐κB could explain the degradation of EG in SIRT1endo‐/‐ mice, resulting in massive shedding of syndecan‐4. Disturbance in blood rheology leading to altered mechanosensory forces felt by EG also leads to the degradation of EG.

## 6 EG AS A POTENTIAL THERAPEUTIC TARGET AGAINST BBB DISRUPTION

The damage of EG destroys the function of BBB, which contributes to a variety of pathologies; therefore, it can be expected that inhibiting EG destruction may improve the pathological state. Considering the influence of EG damage on BBB function, more and more researchers are turning their attention to EG structural protection. Attempts to maintain the integrity of EG can be grouped into two categories: preventing degradation or promoting synthesis. The most widely studied measure to prevent EG shedding has been centered on albumin administration, but beneficial effects from this approach have been observed in only a few animal studies.^82^ Another drug that can prevent EG damage is the TNF‐α inhibitor Etanercept,^83^ but its therapeutic effect has yet to be determined.^84^ Sulodexide is a sulfated polysaccharide complex with vascular‐protective attributes, such as antithrombotic, fibrinolytic, anti‐inflammatory, antioxidant, and anti‐ischemic. In addition, its main mode of action includes restoring HS of EG by inhibiting the activities of heparanase and MMP‐9.^85^ However, no obvious beneficial effect in patients has been identified as of yet.^86^ Another treatment approach is to give a large dose of the steroid hormone methylprednisolone during surgical intervention to reduce the shedding of EG. This treatment method has been successfully applied before neonatal heart surgery.^87^ Hormones play an important role in EG structure protection, although their exact mechanisms remain unclear. The repair of EG may be a selective, effective, and hypothetical goal to change the permeability of the blood‐brain barrier. This will open up a new field for drug intervention for patients with neurological diseases.

## 7 ROLE OF EG INJURY IN NEUROLOGICAL DISORDERS

Many neurological diseases are associated with the accumulation of EG metabolites, such as acetyl heparin sulfate and CS.^88^ Since maintaining EG thickness may be an important aspect of vascular health, reducing EG thickness may lead to vascular disease. In fact, risk factors associated with vascular diseases such as atherosclerosis, hypertension, and diabetes, as well as specific diseases such as luminal cerebral infarction, have been associated with reduced EG thickness. The EG acts as an important component of the BBB, and therefore, impaired EG compromises the BBB, which in turn leads to neurological diseases.^89^ The BBB is essential for maintaining a stable environment within the nervous system, and after BBB disruption, neurotoxic substances, microorganisms, etc., enter the CNS, which can then initiate multiple pathways of neurological disease.^90^, ^91^ Dynamically enhanced cranial magnetic resonance has shown that in early AD,^92^, ^93^ BBB disruption in the hippocampus occurs prior to hippocampal atrophy,^94^ raising the likelihood that BBB disruption precedes neurological degenerative disease. Autopsies have found the presence of blood‐brain barrier disruption in several neurodegenerative diseases such as Alzheimer's disease, Parkinson's disease, and Huntington's disease.

## 8 SUMMARY

The brain contains a variety of balance systems necessary to maintain normal nerve function. In these systems, the BBB plays a central role. EG is an important component that affects various physiological functions of BBB. In the process of disease development, EG, as the outermost layer of the blood vessel wall, will be affected by many inflammatory mediators that can modify and destroy the structure of EG, resulting in endothelial function damage, thus leading to BBB dysfunction^95^ and promoting the occurrence and development of brain diseases. Therefore, monitoring and protecting patients' EG, especially those with chronic diseases, can reduce the occurrence and development of neurological diseases. The association between EG disruption and BBB integrity also reminds researchers and clinicians that EG may be a very valuable clinical target. Restoring or maintaining the structure and function of EG represents an attractive therapeutic strategy, but in order to realize the full potential of this strategy, we need to know more about how the structure of EG responds to disease and the BBB dysfunction after EG injury.

In conclusion, EG, as a key regulator of permeability, cerebral blood flow, capillary perfusion, and cells adhesion, is essential for maintaining brain homeostasis. It is vital to maintain the integrity of EG for the stability of the BBB environment, and we are excited that investigation into the EG as a therapeutic target is just beginning.

## Conflicts of interest

The authors declare no conflicts of interest.

## Author Contributions

Fangfang Zhao wrote the manuscript. Liyuan Zhong revised the manuscript. Yumin Luo designed and critically revised the manuscript.

## Data Availability

Data sharing is not applicable to this article as no datasets were generated or analyzed during the current study.
