# CORRIGENDUM

**DOI:** 10.1111/cns.13652

**Published:** 2021-06-07

Figure 1 of Zhao et al.,^1^ is based on the concept presented in Figure 1 of Haeren et al.,^2^ but failed to cite the latter.

Figure 1 of Zhao et al.^1^ is reproduced below with the appropriate permission and citation:

**FIGURE 1:**
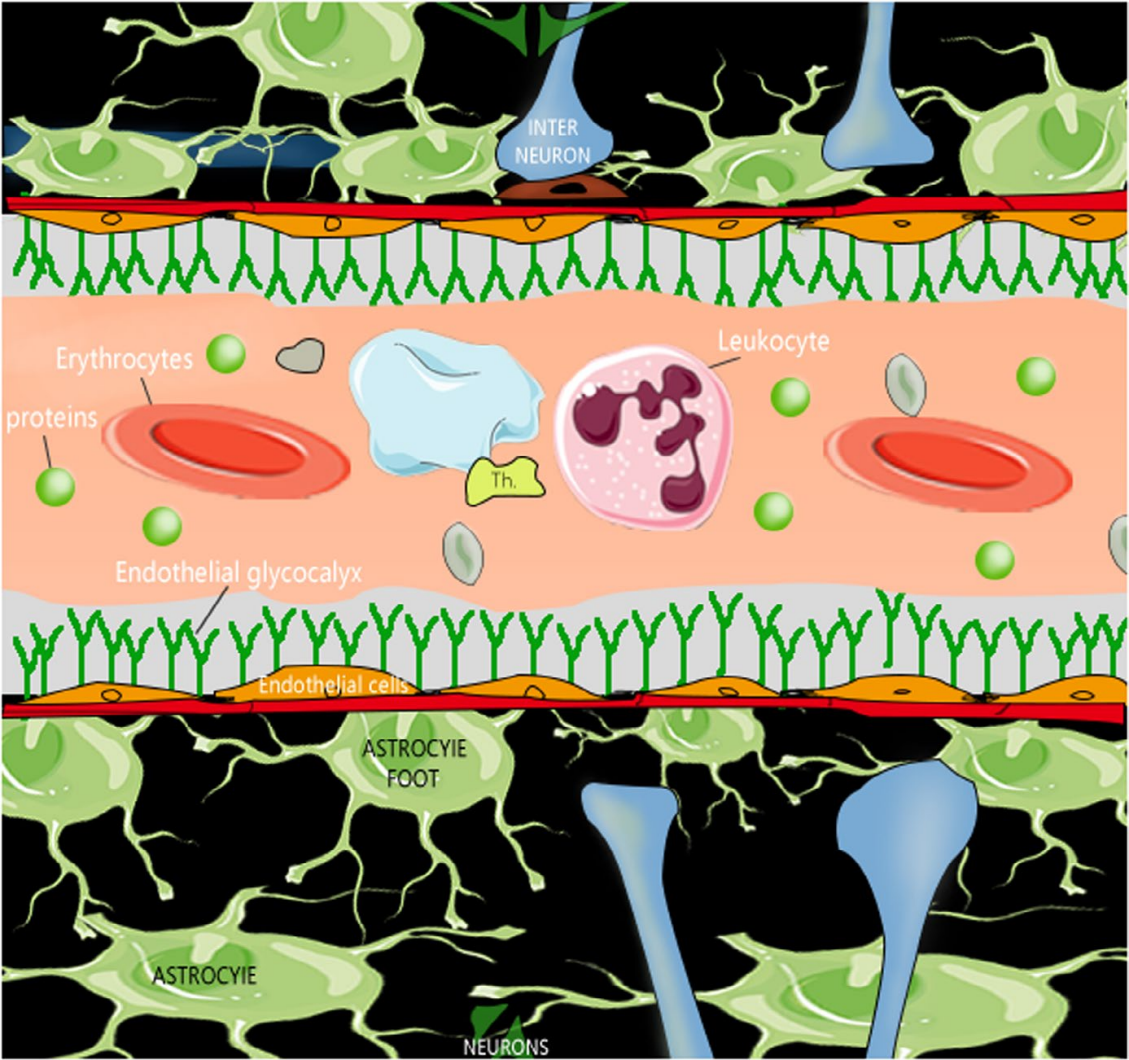
Diagram of the relationship between glycocalyx and the blood‐brain barrier. Republished with permission of Eureka Science (FZC), from Assessment and Imaging of the Cerebrovascular Glycocalyx by Haeren et al.,^2^ *Curr Neurovasc Res*. 2016;13(3):249–60; permission conveyed through Copyright Clearance Center, Inc

We apologize for the error.
